# Vaginal micronized progesterone on preventing luteinizing hormone untimely surge in ART cycles: A prospective proof-of-concept study

**DOI:** 10.5935/1518-0557.20240045

**Published:** 2024

**Authors:** Maria do Carmo Borges de Souza, Roberto de Azevedo Antunes, Marcelo Marinho de Souza, Ana Cristina Allemand Mancebo, Ana Luiza Barbeitas, Veronica de Almeida Raupp, Dandhara Martins Rebello

**Affiliations:** 1 Fertipraxis Centro de Reproducao Humana - Rio de Janeiro (RJ), Brazil

**Keywords:** Vaginal micronized progesterone, progesterone primed cycles, LH surge, oocyte donor program

## Abstract

**Objective:**

A new approach to evaluate whether Progestin-Primed Ovarian Stimulation with micronized vaginal progesterone was as effective as using dydrogesterone in suppress LH pulse surge in young women under stimulation in an oocyte donor programme.

**Methods:**

This prospective study included 21 patients aged 19 to 32 years-old stimulated with Elonva^®^ 150, associated or not with Menopur^®^ or Merional^®^ (75 or 150IU) since the beginning of the cycle, plus HMG 150-225IU after the 8th day or just HMG 150-300IU per day. Patients were placed in a PPOS protocol with micronized vaginal progesterone (MVP) 200 mg (Gynpro^®^ Exeltis or Junno Farmoquimica) every 12 hours or dydrogesterone (Duphaston^®^ Abbott) 10 mg every 8 hours from the start of stimulation until the day after the GnRH trigger with Triptorelin 0.2 mg (Gonapeptyl daily^®^). The primary endpoint was the prevention of untimely LH surge, and secondarily the number of 16 mm follicles, retrieved oocytes and metafase II.

**Results:**

Fourteen oocyte donor patients were prescribed MVP while seven others received dydrogesterone (DYG).The gonadotropin protocols included 04 with Corifollitropin alfa 150 plus HMG since the beginning and complemented after the 7th day, and 17 times of just HMG. There was no diferences in the number of follicles >10≤15mm, ≥16mm or number of metafase II oocytes. There was no untimely LH surge on both groups and no OHSS was developed after the agonist trigger.

**Conclusions:**

Progestin-Primed Ovarian Stimulation with micronized vaginal progesterone seems to be a compelling choice for preventing premature ovulation without compromising oocyte quality in women undergoing ovarian stimulation.

## INTRODUCTION

Several studies have recently suggested that progesterone in Progesterone-Primed Ovarian Stimulation (PPOS) protocols may include a variety of options such as medroxyprogesterone acetate (MPA), dydrogesterone (DYG), or micronized progesterone (MP), all of them being sufficient to prevent an untimely LH rise (Cui *et al*., 2021). This provides a practical and lower cost option than the GnRH antagonist in planned freeze-only cycles, as in preimplantation genetic testing or fertility preservation. Although micronized oral progesterone has been proven effective by Zhu *et al*. (2017) in a prospective noninferiority trial using different doses as well as Ghasemzadeh *et al*. (2020) in a randomized clinical trial, there remain issues related to side effects such as dizziness and sleepiness. Fasting might also contribute to the variable P concentrations observed among patients on oral MP (Simon *et al*., 1993). As Child *et al*. (2018) demonstrated that progesterone can be administered via different routes, such as oral, rectal, vaginal, and intramuscular, with all routes showing a similar efficacy regarding pregnancy, miscarriage, and live birth rate, we decided to investigate the more stable vaginal route of absorption that has no published data yet.

This study aimed to evaluate whether PPOS with micronized vaginal progesterone (MVP) is as effective as using oral dydrogesterone (DYG) to suppress LH pulse surges in young women undergoing stimulation in an egg donor program.

## MATERIAL AND METHODS

This retrospective proof-of-concept study included 21 patients aged 19 to 32 years undergoing ovarian stimulation in an egg donation program in Rio de Janeiro, Brazil. The patients were placed in a PPOS protocol with one of two progesterones: micronized vaginal progesterone 200 mg (Gynpro^®^ Exeltis or Junno Farmoquimica) every 12 hours or dydrogesterone (Duphaston^®^ Abbott) 10 mg every 8 hours from the start of stimulation until the day after the GnRH trigger. Ovarian stimulation was achieved with corifollitropin alfa 150 (Elonva^®^ Organon), associated or not with menotropins (Menopur^®^ Ferring 75 or 150 IU) from the start of the cycle, plus menotropins 150-225 IU after the eighth day, or just menotropins from 150 to 300 IU a day, with doses defined and adjusted based on the clinical decision of the attending physician. Transvaginal sonography was performed on day 2 or 3 of the cycle and then seven days later. The GnRH trigger was performed with Triptorelin 0.2 mg (Gonapeptyl daily^®^ Ferring) when three or more follicles ≥ 17 mm were present. Oocyte retrieval was planned for 36 hours later. The primary endpoint was preventing an untimely LH surge. The secondary endpoint was the number of 16-mm follicles, retrieved oocytes, and the proportion of metaphase II oocytes. Student’s t-test was used to compare numerical variables, and the chi-square test was used to evaluate qualitative data. Differences were considered statistically significant when *p*<.05.

## RESULTS

Fourteen egg donors were prescribed MVP, and seven received DYG. Four patients were treated with corifollitropin alfa 150 plus HMG from the start with complementary therapy after the seventh day, and 17 were given only HMG.

None of the groups had cases of untimely LH surge. The results did not indicate differences in stimulation outcomes (Table 1), and no instances of OHSS were observed after the agonist trigger.

**Table 1:** PPOS cycle results.

Type of progesterone	DYG (07)	MVP (14)	*p* value
**Age**	27.28±3.30	28.28±3.66	0.55
**Follicles > 10 ≤ 15 mm**	10.28±6.67	7.21±3.90	0.19
**Follicles ≥ 16**	15.28±9.58	16.07±6.45	0.84
**Number of oocytes**	23.42±9.58	21.21±9.33	0.68
**Number of Metaphase II**	17.28±10.76	15.21±5.38	0.64

Note: Values are expressed as mean standard deviation.

## DISCUSSION

Kuang *et al*. (2015) were the first progestin-primed ovarian stimulation (PPOS) proponents. It has been proven that PPOS effectively prevents the activation and transmission phases of estradiol-induced LH surges as an alternative to conventional treatment with GnRH antagonists.

Ovarian stimulation protocols with improved efficacy, safety, convenience, and cost have been long sought. Different protocols have been tried involving the choice of specific gonadotropins at individualized doses, mild stimulation, egg freezing and embryo banking options, fertility preservation planning, and use in egg donors. A recent systematic review and meta-analysis by Cui *et al*. (2021) confirmed that PPOS is a valuable tool for fertility preservation cycles, oocyte/embryo banking, PGT-A testing, and, of course, in stimulation protocols for egg donors (Cui *et al*., 2021).

Poor neonatal outcomes from IVF and increases in the prevalence of congenital malformations (Zhang *et al*., 2017) have not been described in PPOS protocols (Huang *et al.,* 2019). The Cochrane Database of Systematic Reviews 2021 (Devall *et al*., 2021) discusses using progestogens to prevent miscarriage. It states that vaginal micronized progesterone may increase the live birth rate for women with a history of one or more miscarriages and early pregnancy bleeding, with likely no difference in adverse events. Due to the lack of trials, the evidence comparing vaginal micronized progesterone versus dydrogesterone was deemed of very low certainty, with effects remaining unclear.

There remains no doubt that MVP or orally administered therapy affects progesterone receptors in the hypothalamus, thus suppressing LH surges. Since both are easy to administer and patient-friendly, patients can choose the treatment of their preference. As Simon *et al*. (1993) indicated, in addition to not causing dizziness and drowsiness as oral MP, MVP avoids the issue of gastric contents interfering with progesterone absorption.

Prospective studies must include larger populations to provide more substantial evidence of the best individual use of micronized progesterone.
